# Clinical and economic burden of pneumococcal disease in US adults aged 19–64 years with chronic or immunocompromising diseases: an observational database study

**DOI:** 10.1186/s12879-018-3326-z

**Published:** 2018-08-29

**Authors:** Dongmu Zhang, Tanaz Petigara, Xiaoqin Yang

**Affiliations:** Center for Observational and Real-World Evidence, Merck & Co., Inc., Kenilworth, NJ 07033 USA

**Keywords:** Pneumonia, Pneumococcal, Pneumococcal infections, Risk factors, Immunocompromised patients, Health resources, Health care costs

## Abstract

**Background:**

Despite the widespread availability of pneumococcal vaccines, rates of pneumococcal disease are disproportionately high in adults with chronic and immunocompromising conditions. This study investigated pneumococcal disease rates and associated resource utilization and costs in this group.

**Methods:**

A retrospective, observational study was conducted using the Truven Health MarketScan® Commercial Claims and Encounters database. The study population was adults aged 19–64 years with continuous health plan enrollment for at least one year before and at least one day after January 1st 2012, 2013 and/or 2014. Medical conditions were identified using ICD-9-CM diagnosis codes and grouped into at-risk (chronic) and high-risk (immunocompromising) conditions. Pneumococcal disease was stratified into all-cause pneumonia (ACP) and invasive pneumococcal disease (IPD).

**Results:**

Thirty-six million adults aged 19–64 years were included in the study. 17% had a condition that put them at increased risk for pneumococcal disease. Rates of ACP and IPD in adults with at-risk conditions were 3.6 and 4.6 times the rate in healthy adults, respectively, and 5.3 and 10.5 for adults with high-risk conditions. Risk was particularly high in adults with ≥2 medical conditions: rates of ACP and IPD were 8.1 and 10.6 times higher in adults with at-risk conditions than healthy adults and 6.3 and 13.4 times higher in adults with high-risk conditions, respectively. Resource use and costs were substantially higher per episode of ACP in at-risk and high-risk adults, with costs reaching $6,534 and $9,168, compared to $4,725 for healthy adults.

**Conclusions:**

Pneumococcal disease rates in at-risk and high-risk adults are significantly higher than healthy adults leading to substantial economic burden.

**Electronic supplementary material:**

The online version of this article (10.1186/s12879-018-3326-z) contains supplementary material, which is available to authorized users.

## Background

*S. pneumoniae,* is a gram-positive bacterium that colonizes the upper respiratory tract [1]. Infection can be categorized as invasive or non-invasive depending on the manifestation [2]. Invasive pneumococcal disease (IPD) comprises bacteremic pneumococcal pneumonia, meningitis and bacteremia without focus, whereas non-invasive disease includes non-bacteremic pneumococcal pneumonia, otitis media, sinusitis and bronchitis [2, 3].

IPD is a leading cause of morbidity and mortality [4], particularly in adults aged 18–64 years with chronic or immunocompromising conditions and those aged 65 and over [5]. In the United States (US) in 2015, 52% of the 29,500 IPD cases and 40% of the 3350 IPD-related deaths occurred in adults aged 18–64 years [5]. The associated economic burden is also substantial: it is estimated that hospitalizations for pneumococcal pneumonia will double between 2004 and 2040 and the total economic burden will increase by $2.5 billion annually [6].

Adults with underlying chronic conditions such as diabetes, lung disease, heart disease or liver disease, are considered at increased risk of developing pneumococcal disease, with reported incidence of up to 483 per 100,000 person-years [7]. This is particularly pertinent, given that by 2025, chronic diseases are estimated to affect 164 million adults in the US – ~ 49% of the current population [8]. Pneumococcal disease incidence in adults with immunocompromising conditions (such as human immunodeficiency virus [HIV] and chronic renal disease) is even higher, with incidence rates reaching as high as 2031 per 100,000 person-years [7].

Currently, two pneumococcal vaccines are approved for use in adults in the US: a pneumococcal conjugate vaccine (PCV13) and a pneumococcal polysaccharide vaccine (PPSV23) [9]. Since 1997, the Advisory Committee on Immunization Practices (ACIP) has recommended that adults under 65 years with medical conditions such as chronic heart disease, chronic lung disease, or diabetes, among others, receive the PPSV23 vaccination [10]. Since 2012, ACIP has recommended PCV13 for adults aged ≥19 years with immunocompromising conditions followed by PPSV23 8 weeks later [11]. However, despite widespread availability of pneumococcal vaccines in the US, rates of IPD remain disproportionately high in adults with chronic medical or immunocompromising conditions, including those with ≥2 conditions [12, 13]. Pneumococcal vaccination coverage in 2015 among adults aged 19–64 years at increased risk for pneumococcal disease was only 23.0% [14], substantially below the US Healthy People 2020 objective of at least 60% immunization coverage in adults aged 18–64 years at increased risk of pneumococcal disease [15].

In the US, Weycker et al. (2016) conducted a retrospective cohort study (2007–2010), in which they reported the rates and costs of IPD and all-cause pneumonia (ACP) in adults with chronic medical and immunocompromising conditions [16]. Rates and costs of IPD and ACP were consistently higher among these adults versus their healthy counterparts [16]. The objective of our study was to provide updated estimates of pneumococcal disease rates, and to provide more detailed estimates of healthcare resource utilization and costs in US adults aged 19–64 years with chronic and immunocompromising conditions recommended for pneumococcal vaccination by the ACIP.

## Methods

### Study design and data source

This was a retrospective, observational cohort study which used the Truven Health MarketScan® Commercial Claims and Encounters database. This database provides patient demographics, health plan information, medical diagnosis/procedure codes, prescriptions, healthcare utilization and cost data. The database represents approximately 100 employer-sponsored private health plans covering approximately 45 million members. Each member in the database has a unique identifier that can be used to track adults across sites of service and providers over time.

### Study sample

Adults who were 19–64 years old between 2012 and 2014, and who had continuous health plan enrollment (no gap of > 45 days) for at least one year before and at least one day after January 1st, 2012, 2013 and/or 2014 were included. The analysis was conducted in 2017.

Medical conditions were identified using ICD-9-CM (International Classification of Diseases, Ninth Revision, Clinical Modification) diagnosis/procedure codes in medical claims (both inpatient and outpatient) during the one year prior to January 1st of 2012, 2013, or 2014. Two ICD-9-CM codes were required to identify an individual as having a particular condition (Additional file 1: Table S1). Medical conditions were grouped into at-risk (chronic) conditions and high-risk (immunocompromising) conditions separately. At-risk conditions included: chronic heart disease, asthma, chronic lung disease, diabetes mellitus, and chronic liver disease. High-risk conditions included: chronic renal disease, cancer, functional or anatomic asplenia, HIV, and organ transplantation. If an adult had both an at-risk and a high-risk condition, he/she was assigned to the high-risk condition only. If an adult had ≥2 high-risk conditions, he/she was assigned to each high-risk condition identified. If an adult had ≥2 at-risk conditions and no high-risk condition, he/she was assigned to each at-risk condition identified. Adults without evidence of any of the aforementioned conditions in the one year pre-period were classified as healthy.

Episodes of pneumococcal disease that occurred during each study year (January 1st to December 31st) were identified based on ICD-9-CM diagnosis codes (Additional file 1: Table S1). Episodes of pneumococcal disease were considered as separate events if they occurred ≥90 days apart. Pneumococcal diseases included in the study were stratified into ACP and IPD. Similar to previous publications, ACP was selected as an alternative to pneumococcal pneumonia because the latter is known to be under-reported in healthcare claims. However, a large proportion (27–31%) of ACP has been attributed to *S. pneumoniae* [16–18].

### Study variables

Pneumococcal disease-related healthcare resource utilization was computed based on medical claims submitted during each pneumococcal disease episode. Healthcare resources included office visits, emergency department (ED) visits, inpatient hospitalizations, and length of hospital stay. Pneumococcal disease-related healthcare costs were calculated as the total payment (amount reimbursed by health plans and paid out-of-pocket by adults) on all claims submitted during the pneumococcal disease episode. The rate of pneumococcal diseases was expressed as the number of cases per 100,000 person-years, and calculated as the number of pneumococcal disease episodes divided by the sum of person-years at-risk.

Sociodemographic characteristics of the study cohort were reported as person-years instead of number of persons. This allowed adults who met criteria for inclusion in multiple calendar years and/or who had ≥2 conditions to contribute data to each yearly cohort for which they were eligible. Sociodemographic characteristics included: age group (19–49; 50–64 years), gender and health plan type (Fee-for-Service; Preferred/Exclusive Provider Organization [PPO/EPO]; Health Maintenance Organization [HMO]; Point of Service [POS]; High Deductible Consumer Driven Health Plan [HDHP/CDHP]; unknown) (Additional file 1: Table S2).

### Statistical analysis

Descriptive statistics including frequencies and percentages for categorical variables and mean (SD) for continuous variables were calculated to describe the characteristics of the cohort. Rates and incidence rate ratios (IRRs) (95% confidence intervals [CIs]) of pneumococcal diseases were reported among patients with each at-risk or high-risk condition compared with their healthy counterparts. Mean (SD) of healthcare resource utilization and costs per episode of pneumococcal disease among patients with at least one episode of pneumococcal disease were also reported. T-test was used to compare healthcare resource utilization and costs between patients with chronic or immunocompromising conditions, and healthy people. All statistical analyses were performed using SAS version 9.3 (SAS Institute, Cary, NC).

## Results

### Demographics

Cohort construction by year is reported in Table 1. Approximately 51 million adults aged 19–64 years were identified from 2012 to 2014, of which approximately 36 million were eligible for inclusion in the study.

**Table 1:** Cohort construction

Criteria	Number of unique adults			
	2012	2013	2014	Total
Aged 19–64 years	33,431,239	27,591,388	29,913,349	50,807,486
Had continuous health plan enrollment (no gap of > 45 days) for at least one year (365 days) before January 1st and at least one day since January 1st	24,402,729	18,802,142	20,338,835	35,696,718

Full demographics are reported in Table 2. Most adults were aged 19–49 years (62%); 48% were female. The most commonly reported health plan type was PPO/EPO (63%). Among the population aged 19–64 years, 17% had an at-risk or high-risk condition. Among those with at-risk conditions, the most frequent condition was diabetes mellitus (51%) followed by asthma (22%). Among those with high-risk conditions, the most frequent condition was cancer (70%), followed by organ transplant (20%). Most adults with at-risk conditions had only one condition (86%). Most adults with high-risk conditions had only one condition (89%).

**Table 2:** Cohort characteristics

Variable	Person-Years	Percent
Age group		
19–49 years	35,360,219.65	61.92
50–64 years	21,748,096.07	38.08
Gender		
Female	27,290,491.17	47.79
Male	29,817,824.55	52.21
Health plan type		
Fee-for-service	1,159,288.49	2.03
PPO/EPO	36,123,603.49	63.25
HMO	6,943,639.48	12.16
POS	4,046,790.82	7.09
HDHP/CDHP	6,592,500.88	11.54
Unknown	2,242,492.56	3.93
Medical conditions		
Healthy	47,370,548.67	82.95
With at-risk or high-risk conditions	9,737,767.05	17.05
At-risk conditions		
Overall	7,119,125.33	100.0
Asthma	1,596,160.50	22.42
Chronic heart disease	1,432,517.29	20.12
Chronic lung disease	1,007,145.18	14.15
Chronic liver disease	607,949.48	8.54
Diabetes mellitus	3,656,304.75	51.36
Number of at-risk conditions		
1	6,089,016.03	85.53
≥ 2	1,030,109.30	14.48
High-risk conditions		
Overall	2,618,641.72	100.0
Chronic renal disease	424,677.27	16.22
Cancer	1,825,298.26	69.70
Asplenia	40,691.59	1.55
HIV	108,115.60	4.13
Organ transplant	522,834.47	19.97
Number of high-risk conditions		
1	2,325,408.37	88.80
≥ 2	293,233.35	11.19
Number of years in follow-up period		
Mean (SD)	0.93 (0.20)	

*HDHP/CDHP* High Deductible/Consumer Driven Health Plan, *HIV* human immunodeficiency virus, *HMO* Health Maintenance Organization, *POS* Point of Service, *PPO/EPO* Preferred/Exclusive Provider Organization, *SD* standard deviation

### Rates of ACP and IPD

Table 3 presents the rates of ACP and IPD in the study population. In at-risk adults, rates of ACP and IPD were approximately 3.7 and 4.7 times the rates in healthy adults, respectively. Rates of ACP and IPD for adults with 1 at-risk condition were 2.9 and 3.7 times higher than healthy adults, but 8.1 and 10.6 times higher for adults with ≥2 at-risk conditions. Adults with chronic lung disease were at the greatest risk of both ACP and IPD, compared to healthy adults (IRR = 8.2, IRR = 10.6, respectively). The risk of ACP in adults with chronic heart disease was 5.5 times the risk compared to healthy adults, followed by asthma (IRR = 4.2), chronic liver disease (IRR = 3.7) and diabetes mellitus (IRR = 3.3). The risk of IPD in adults with chronic liver disease was 7.2 times the risk in healthy adults, followed by chronic heart disease (IRR = 6.0), diabetes mellitus (IRR = 4.7) and asthma (IRR = 4.0).

**Table 3:** Rates of ACP and IPD

		ACP		IPD	
Variable	Person-Years	Rate per 100, 000 person-years	Rate ratio (95% CI)	Rate per 100, 000 person-years	Rate ratio (95% CI)
Overall	57,108,315.72	656.40	–	3.62	–
Age group					
19–49	35,360,219.65	438.26	–	1.77	–
50–64	21,748,096.07	1011.08	–	6.63	–
Gender					
Female	27,290,491.17	658.00	–	3.77	–
Male	29,817,824.55	654.93	–	3.48	–
Health plan type					
Fee-for-service	1,159,288.49	1395.17	–	10.44	–
PPO/EPO	36,123,603.49	646.15	–	3.58	–
HMO	6,943,639.48	620.81	–	3.25	–
POS	4,046,790.82	705.74	–	3.56	–
HDHP/CDHP	6,592,500.88	566.14	–	2.76	–
Unknown	2,242,492.56	726.11	–	4.50	–
Medical conditions					
Healthy	47,370,548.67	429.65	1.00	1.91	1.00
Any at-risk or high-risk condition	9,737,767.05	1759.46	4.10 (4.07–4.12)	11.92	6.23 (5.72–6.80)
At-risk conditions					
Overall	7,119,125.33	1573.40	3.66 (3.64–3.69)	8.91	4.66 (4.21–5.15)
Asthma	1,596,160.50	1783.09	4.15 (4.10–4.20)	7.71	4.03 (3.34–4.86)
Chronic heart disease	1,432,517.29	2343.78	5.46 (5.39–5.52)	11.38	5.95 (5.04–7.03)
Chronic lung disease	1,007,145.18	3530.77	8.22 (8.13–8.31)	20.16	10.54 (9.13–12.33)
Chronic liver disease	607,949.48	1603.75	3.73 (3.66–3.81)	13.82	7.22 (5.78–9.03)
Diabetes mellitus	3,656,304.75	1396.74	3.25 (3.22–3.28)	9.03	4.72 (4.16–5.35)
Number of at-risk conditions					
1	6,089,016.03	1251.81	2.91 (2.89–2.94)	6.98	3.65 (3.25–4.10)
≥ 2	1,030,109.30	3474.29	8.09 (8.00–8.18)	20.29	10.61 (9.13–12.33)
High-risk conditions					
Overall	2,618,641.72	2265.30	5.27 (5.22–5.32)	20.12	10.52 (9.45–11.72)
Chronic renal disease	424,677.27	4045.19	9.42 (9.27–9.56)	41.44	21.67 (18.44–25.47)
Cancer	1,825,298.26	2132.14	4.96 (4.91–5.02)	18.79	9.83 (8.68–11.13)
Asplenia	40,691.59	3801.77	8.85 (8.42–9.30)	22.12	11.56 (6.00–22.30)
HIV	108,115.60	1748.13	4.07 (3.89–4.26)	33.30	17.41 (12.48–24.29)
Organ transplant	522,834.47	1611.03	3.75 (3.67–3.83)	8.03	4.20 (3.08–5.72)
Number of high-risk conditions					
1	2,325,408.37	2209.68	5.14 (5.09–5.19)	19.44	10.16 (9.08–11.38)
≥ 2	293,233.35	2706.38	6.30 (6.16–6.44)	25.58	13.37 (10.57–16.92)

*ACP* all-cause pneumonia, *HDHP/CDHP* High Deductible/Consumer Driven Health Plan, *CI* confidence interval, *HIV* human immunodeficiency virus, *HMO* Health Maintenance Organization, *IPD* invasive pneumococcal disease, *POS* Point of Service, *PPO/EPO* Preferred/Exclusive Provider Organization

Among high-risk adults, rates of ACP and IPD were approximately 5.3 and 10.5 times the rates in healthy adults respectively. Rates of ACP and IPD for adults with 1 high-risk condition were 5.1 and 10.2 times the rates for healthy adults, but 6.3 and 13.4 times the rates for adults with ≥2 high-risk conditions respectively. Adults with chronic renal disease were at the greatest risk of both ACP and IPD, compared to healthy adults (IRR = 9.4, IRR = 21.7, respectively). The risk of ACP in adults with asplenia was 8.9 times the risk among healthy adults, followed by cancer (IRR = 5.0), HIV (IRR = 4.1), and organ transplant (IRR = 3.8). The risk of IPD in adults with HIV was 17.4 times the risk in healthy adults, followed by asplenia (IRR = 11.6), cancer (IRR = 9.8) and organ transplant (IRR = 4.2).

### Resource use associated with ACP and IPD

Table 4 shows healthcare resource utilization per ACP and IPD episode. Per ACP episode, resource use was significantly higher for adults with at-risk conditions compared to healthy adults, with the exception of office visits which were significantly lower (all *P* < 0.05). Per IPD episode, resource use was similar for adults with at-risk conditions and their healthy counterparts across all measures studied (all *P* > 0.05).

**Table 4:** Healthcare resource utilization per episode of ACP and IPD

	Resource use							
	Number of office visits (mean [SD])		Number of emergency department visits (mean [SD])		Number of inpatient hospital visits (mean [SD])		Length of inpatient hospital stay (mean [SD])	
	ACP	IPD	ACP	IPD	ACP	IPD	ACP	IPD
Healthy	0.58 (0.76)	0.10 (0.52)	0.22 (0.43)	0.40 (0.51)	0.35 (0.52)	0.96 (0.27)	1.43 (4.09)	7.50 (8.09)
At risk conditions								
Overall	0.43 (0.75)†	0.08 (0.55)	0.23 (0.46)†	0.40 (0.52)	0.54 (0.59)†	0.96 (0.25)	2.41 (5.40)†	7.64 (7.93)
Asthma	0.49 (0.80)†	0.10 (0.58)	0.25 (0.47)†	0.45 (0.52)	0.45 (0.57)†	0.95 (0.22)	1.98 (4.68)†	7.18 (7.71)
Chronic heart disease	0.32 (0.69)†	0.06 (0.56)	0.22 (0.46)	0.42 (0.54)	0.68 (0.61)†	0.97 (0.23)	3.04 (6.05)†	8.31 (8.81)
Chronic lung disease	0.39 (0.76)†	0.07 (0.65)	0.25 (0.48)†	0.43 (0.54)	0.59 (0.62)†	0.95 (0.26)	2.88 (6.04)†	7.06 (6.73)
Chronic liver disease	0.37 (0.75)†	0.21 (1.18)	0.23 (0.47)*	0.37 (0.49)	0.62 (0.66)†	0.99 (0.19)	2.90 (6.71)†	7.15 (7.61)
Diabetes mellitus	0.41 (0.71)†	0.06 (0.41)	0.23 (0.45)*	0.39 (0.51)	0.56 (0.60)†	0.95 (0.27)	2.49 (5.62)†	7.88 (8.36)
High-risk conditions								
Overall	0.29 (0.67)†	0.08 (0.40)	0.24 (0.49)†	0.34 (0.53)*	0.73 (0.66)†	0.98 (0.31)	3.46 (6.56)†	7.78 (8.03)
Chronic renal disease	0.21 (0.61)†	0.07 (0.33)	0.26 (0.51)†	0.30 (0.46)*	0.79 (0.65)†	0.99 (0.30)	3.82 (7.24)†	8.38 (8.49)
Cancer	0.30 (0.68)†	0.08 (0.35)	0.23 (0.50)†	0.35 (0.56)	0.74 (0.68)†	0.99 (0.34)	3.48 (6.37)†	7.68 (8.11)
Asplenia	0.19 (0.60)†	0.89 (1.62)	0.24 (0.47)	0.22 (0.44)	0.85 (0.63)†	0.89 (0.93)	4.03 (7.76)†	6.11 (7.44)
HIV	0.32 (0.64)†	0.08 (0.37)	0.28 (0.49)†	0.36 (0.49)	0.60 (0.61)†	1.00 (0.00)†	3.02 (6.33)†	7.06 (8.62)
Organ transplant	0.38 (0.77)†	0.05 (0.22)	0.23 (0.46)	0.48 (0.86)	0.63 (0.65)†	0.95 (0.44)	2.90 (6.25)†	7.14 (6.76)

*P < 0.05 (comparisons between at-risk or high-risk adults and healthy adults)

†*P* < 0.0001 (comparisons between at-risk or high-risk adults and healthy adults)

*ACP* all-cause pneumonia, *HIV* human immunodeficiency virus, *IPD* invasive pneumococcal disease, *SD* standard deviation

Per ACP episode, resource use was significantly higher for adults with high-risk conditions compared to their healthy counterparts, with the exception of office visits, which was significantly lower (all *P* < 0.0001). Per IPD episode, resource use was similar for adults with high-risk conditions compared to their healthy counterparts (*P* > 0.05), with the exception of adults with chronic renal disease who had a significantly lower number of ED visits, and adults with HIV who had a significantly higher number of inpatient hospitalizations (both *P* < 0.05).

Table 5 shows healthcare resource utilization per ACP and IPD episode in adults with ≥2 at-risk or high-risk conditions. Per ACP episode, resource use was higher in those with ≥2 at-risk or high-risk conditions across all measures except for office visits. Per IPD episode, no statistically significant differences were observed in those with ≥2 at-risk or high-risk conditions in all measures as compared to healthy adults.

**Table 5:** Healthcare resource utilization per episode of ACP and IPD by number of conditions

	Resource use							
	Number of office visits (mean [SD])		Number of emergency department visits (mean [SD])		Number of inpatient hospital visits (mean [SD])		Length of inpatient hospital stay (mean [SD])	
	ACP	IPD	ACP	IPD	ACP	IPD	ACP	IPD
Healthy	0.58 (0.76)	0.10 (0.52)	0.22 (0.43)	0.40 (0.51)	0.35 (0.52)	0.96 (0.27)	1.43 (4.09)	7.50 (8.09)
At risk conditions								
Overall	0.43 (0.75)†	0.08 (0.55)	0.23 (0.46)†	0.40 (0.52)	0.54 (0.59)†	0.96 (0.25)	2.41 (5.40)†	7.64 (7.93)
1	0.47 (0.75)†	0.07 (0.42)	0.23 (0.45)*	0.38 (0.50)	0.50 (0.58)†	0.97 (0.25)	2.11 (4.80)†	7.66 (7.85)
≥2	0.34 (0.73) †	0.10 (0.76)	0.24 (0.48)*	0.44 (0.54)	0.65 (0.62) †	0.95 (0.24)	3.04 (6.45) †	7.60 (8.13)
High-risk conditions								
Overall	0.29 (0.67)†	0.08 (0.40)	0.24 (0.49)†	0.34 (0.53)*	0.73 (0.66)†	0.98 (0.31)	3.46 (6.56)†	7.78 (8.03)
1	0.36 (0.70)†	0.06 (0.34)	0.22 (0.46)	0.34 (0.50)	0.63 (0.63)†	0.97 (0.28)	2.79 (5.50)†	7.19 (7.14)
≥2	0.25 (0.65) †	0.09 (0.42)	0.25 (0.51) †	0.34 (0.54)	0.78 (0.67) †	0.99 (0.33)	3.79 (6.99)†	8.00 (8.34)

*P < 0.05 (comparisons between at-risk or high-risk adults and healthy adults)

†*P* < 0.0001 (comparisons between at-risk or high-risk adults and healthy adults)

*ACP* all-cause pneumonia, *HIV* human immunodeficiency virus, *IPD* invasive pneumococcal disease, *SD* standard deviation

### Costs associated with ACP and IPD

**Fig. 1:**
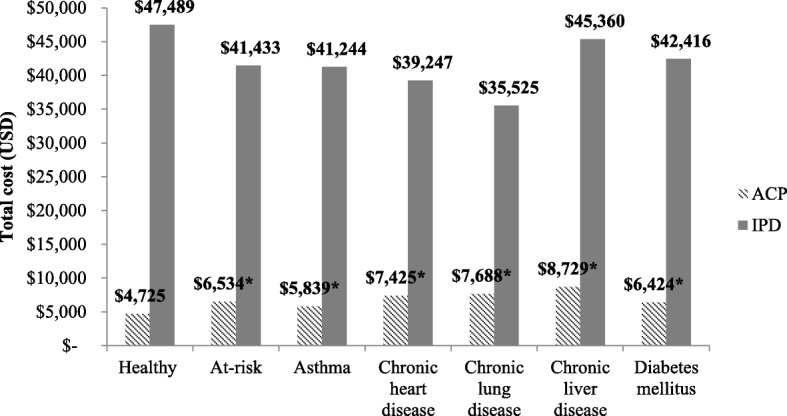
Mean cost per episode of ACP and IPD in healthy and at-risk adults 19–64 years. *P < 0.0001. *ACP* all-cause pneumonia, *IPD* invasive pneumococcal disease

Figure 1 presents the average cost per episode of ACP and IPD in healthy and at-risk adults aged 19–64 years, respectively. Average cost per ACP episode in at-risk adults overall ($6,534) were significantly higher than in healthy adults ($4,725) (*P* < 0.0001), with the highest costs seen in adults with chronic liver disease ($8,729), followed by chronic lung disease ($7,688), and chronic heart disease ($7,425). For IPD, the average cost per IPD episode in adults with at-risk conditions was similar to that of healthy adults across all conditions (*P* > 0.05).

**Fig. 2:**
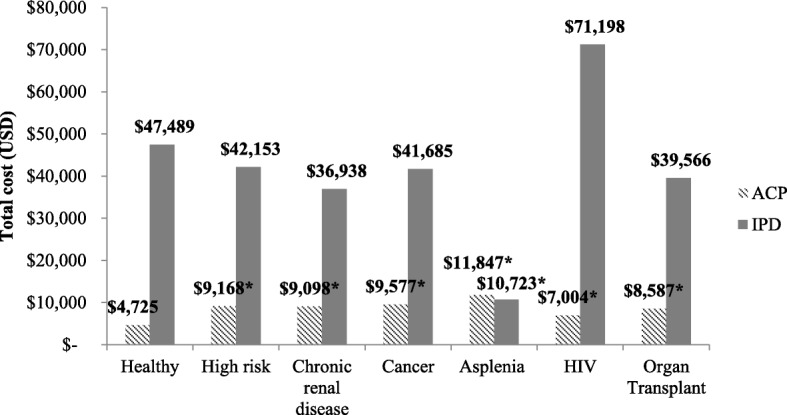
Mean cost per episode of ACP and IPD in healthy and high-risk adults 19–64 years. *P **<** 0.0001. *ACP* all-cause pneumonia, *IPD* invasive pneumococcal disease

Figure 2 presents the average cost per episode of ACP and IPD in healthy and high-risk adults aged 19–64 years, respectively. Costs per ACP episode in high-risk adults overall ($9,168) were significantly higher than in healthy adults ($4,725) (*P* < 0.0001), with the highest costs seen in adults with asplenia ($11,847), followed by cancer ($9,577), and chronic renal disease ($9,098). The average cost per IPD episode was similar between adults with high-risk conditions and healthy adults, with the exception of asplenia, which had significantly lower costs ($10,723 versus $47,489; *P* < 0.0001).

Costs per ACP episode were significantly higher in adults with either 1 or ≥ 2 at-risk or high-risk conditions than in healthy adults (*P* < 0.0001). Adults with ≥2 at-risk or high-risk conditions had higher costs per ACP episode than those with only one condition (at-risk: $7,796 versus $5,940; high-risk: $9,761 versus $7,956, respectively). Costs per IPD episode were similar in adults with either 1 or ≥ 2 conditions compared to healthy adults. Adults with ≥2 at-risk conditions had lower costs per IPD episode than adults with only one at-risk condition ($38,638 versus $42,807, respectively), but adults with ≥2 high-risk conditions had higher costs per IPD episode than adults with only one high-risk condition ($42,393 versus $41,515, respectively).

## Discussion

This study examined IPD and ACP rates in adults aged 19–64 years with at-risk or high-risk conditions during 2012–2014. Pneumococcal disease rates in these populations were lower for each at-risk and high-risk condition compared to a similar retrospective study conducted by Weycker et al. (2016) for the period 2007–2010. These findings may reflect indirect effects from the infant 13-valent pneumococcal conjugate vaccine program, which was implemented in the United States in 2010 [19]. Pneumococcal vaccine coverage in at-risk and high-risk adults 19–64 years remained constant during the study time period at approximately 20% [14].

Our findings, however, continue to indicate that pneumococcal disease rates are substantially higher among adults aged 19–64 years with at-risk or high-risk conditions compared to healthy adults of the same age, with rates of disease particularly high in those with ≥2 at-risk or high-risk conditions. Incidence rate ratios remain consistent with those observed between 2007 to 2010 by Weycker et al. (2016) in at-risk and high-risk adults, who reported ACP rates of 3.6 and 6.8 and IPD rates of 3.4 and 9.7 times the rate of healthy adults [16]. Weycker et al. (2016) also reported rates of pneumococcal disease to increase with an increasing number of conditions [16].

Healthcare resource use per ACP episode, including ED and inpatient visits, was generally higher in both at-risk and high-risk conditions compared to healthy patients, but healthy patients had more office visits. This may reflect greater severity of the ACP episode in at-risk and high-risk adults compared to healthy adults. However, resource use per IPD episode for at-risk and high-risk adults was generally similar, or lower than, healthy adults. This is consistent with previous findings by Weycker et al. (2016) in which lower costs per IPD episode in at-risk and high-risk adults (18–64 years) compared to healthy adults were reported, and is likely due to the small number of IPD episodes included in these studies [16].

Unexpectedly, adults with asplenia had significantly lower costs per IPD episode compared to healthy adults. One study found that asplenic patients were more likely to experience a more severe pneumococcal infection requiring mechanical ventilation and ICU admission compared to patients with a spleen [20]. On the other hand, our study found that asplenic IPD patients had, on average, more outpatient visits, fewer inpatient episodes and a shorter inpatient length of stay compared to their healthy counterparts. This may reflect early treatment of asplenic patients, who are recommended to receive immediate antibiotic therapy for fever. However, further research is required to corroborate this finding as only a relatively small proportion of adults with asplenia were included in this study - only 1.6% of adults with high-risk conditions.

However, although adults aged 19–64 years with chronic and immunosuppressive conditions are at increased risk for pneumococcal disease, vaccine uptake in this population remains well below the Healthy People 2020 target of 60% (approximately 20% between 2012 and 2014, rising to 23% and 24% in 2015 and 2016 respectively) [14, 15]. A recent article also demonstrated that, despite ACIP recommendations, as few as 8% of adults newly diagnosed with a chronic condition receive pneumococcal vaccination within a year of diagnosis [21]. The low coverage is particularly concerning given the high burden of pneumococcal disease identified in the present analysis [22].

### Limitations

Several limitations inherent to administrative claims data apply to our study. Since chronic and immunosuppressive conditions were identified based on ICD-9-CM diagnosis/procedure codes, coding inaccuracies may lead to misclassification. Pneumococcal disease is also typically under-coded in claims, which may have led to an underestimation of the disease burden. Finally, although managed care is the predominant form of healthcare care in the US, the inclusion of mainly US managed care enrollees may limit generalization outside of this population.

## Conclusion

Adults aged 19–64 years with at-risk and high-risk conditions have significantly higher rates of pneumococcal disease compared to healthy adults. Costs per episode of ACP are also significantly higher in adults with at-risk and high-risk conditions.

## Additional file

Additional file 1: **Table S1**. ICD codes. Description: A list of ICD-9-CM diagnosis and procedure codes, which were used to identify and group medical conditions into high-risk and at-risk conditions. **Table S2**. Title: Health plan descriptions. Description: US plan types included in the study, and accompanying descriptions. (DOCX 311 kb)

## Abbreviations

ACIP: Advisory Committee on Immunization Practices
ACP: All-cause pneumonia
ED: Emergency department
HDHP/CDHP: High Deductible Consumer Driven Health Plan
HIV: Human immunodeficiency virus
HMO: Health Maintenance Organization
IPD: Invasive pneumococcal disease
IRB: Institutional Review Board
IRR: Incidence rate ratio
NFID: National Foundation for Infectious Disease
PCV13: Pneumococcal conjugate vaccine
POS: Point of Service
PPO/EPO: Preferred/Exclusive Provider Organization
PPSV23: Pneumococcal polysaccharide vaccine
SD: Standard deviation
US: United States
