# Supplementary material for: Clinical and economic burden of pneumococcal disease in US adults aged 19–64 years with chronic or immunocompromising diseases: an observational database study
Source: BMC Infect Dis. 2018 Aug 29;18:436. doi: 10.1186/s12879-018-3326-z (PMC6116536; doi:10.1186/s12879-018-3326-z)
Supplement: Supplementary file 1 — Table S1. ICD codes. Description: A list of ICD-9-CM diagnosis and procedure codes, which were used to identify and group medical conditions into high-risk and at-risk conditions. Table S2. Title: Health plan descriptions. Description: US plan types included in the study, and accompanying descriptions. (DOCX 311 kb) [file 12879_2018_3326_MOESM1_ESM.docx]

**Supplementary materials**

Table 1. ICD codes

| **Chronic medical conditions** | **ICD-9-CM diagnosis codes** | **ICD-9-CM procedure codes** | **CPT4 procedure codes** |
| --- | --- | --- | --- |
| Chronic heart disease | 393.xx-398.xx, 402.01, 402.11, 402.91, 404.01, 404.03, 404.11, 404.13, 404.91, 404.93, 413.xx, 414.xx, 416.xx, 425.xx, 428.xx, 429.xx, 746.xx | N/A | N/A |
| Asthma | 493.xx | N/A | N/A |
| Chronic lung disease | 490.xx–492.xx, 494.xx–496.xx, 500.xx–505.xx, 515.xx, 516.xx, 518.83, 518.89 | N/A | N/A |
| Diabetes | 249.xx, 250.xx | N/A | N/A |
| Chronic liver disease | 070.2x, 070.3x, 070.44, 070.54, 070.70, 070.71, 571.0x, 571.2x–571.9x, 572.xx, 573.xx | N/A | N/A |
| Asplenia | 282.4x, 282.6x, 759.0x, 289.4x, 289.5x | N/A | 38100, 38101, 38102, 38115, 38120, 38129 |
| HIV | 042.xx, V08.xx, 079.53 | N/A | N/A |
| Chronic renal disease | 403.xx, 404.02, 404.12, 404.92, 581.xx, 582.xx, 585.xx, 586.xx | N/A | N/A |
| Cancer | 140.xx-209.xx, 230.xx–234.xx | N/A | N/A |
| Organ transplantation | N/A | 0091x, 0092x, 0093x, 0794x, 3751x, 4697x, 5553x, 5569x, 116xx, 335xx, 336xx, 410xx, 504xx, 505xx, 528xx | '00144', '00580', '00796', '00868', '29868', '32851', '32852', '32853', '32854', '32855', '32856', '33933', '33935', '33944', '33945', '38240', '38241', '38242', '44135', '44136', '44137', '44715', '44720', '44721', '47135', '47136', '47143', '47144', '47145', '47146', '47147', '48551', '48552', '48554', '48556', '50323', '50325', '50327', '50328', '50329', '50340', '50360', '50365', '50370', '65710', '65730', '65750', '65755', '65756', '65757', '65780', '65781', '76776', '76778', '81267', '81268' |
| IPD – bacteremia | 790.7x, 771.83 + 041.2x, 038.2x, 038.9x + 041.2x | N/A | N/A |
| IPD – meningitis | 320.1x, 320.9x + 041.2x, 322.9x + 041.2x | N/A | N/A |
| IPD - other | 421.xx + 041.2x, 711.0x + 041.2x, 730.0x, 730.2x + 041.2x | N/A | N/A |
| All-cause pneumonia | 480.xx–486.xx, 487.0x, 510.xx, 511.1x, 511.9x |  |  |
| Pneumococcal pneumonia | 481.xx, 482.9x + 041.2x, 485.xx + 041.2x, 486.xx + 041.2x, 510.xx + 041.2x, 511.1x, 511.9x + 041.2x |  |  |

CM = clinical modification; CPT4 = Current Procedural Terminology, 4th Edition; HIV = human immunodeficiency virus;
 ICD = International Classification of Diseases; IPD = invasive pneumococcal disease.

Table 2. Health plan descriptions^1^

| **Plan type** | **Description** |
| --- | --- |
| Traditional fee-for-service plan | This type of plan finances, but does not deliver, health care services; the plan allows participants the choice of any provider, without affecting reimbursement. |
| Exclusive provider organization (EPO) | This type of plan obligates employees to use only the plan’s providers in order to receive coverage, in contrast to PPO benefit plans, which merely offer a financial incentive for enrollees to use the preferred provider. |
| Preferred provider organization (PPO) | This type of plan provides coverage through a network of participating health care providers. Enrollees may receive services outside the network, but generally at higher costs. The additional costs may be in the form of higher deductibles, higher coinsurance rates, or both, or in the form of non-discounted charges from providers. |
| Point-of-service (POS) plan | This type of plan provides services through a network of participating health care providers. Services received within the network or through select medical facilities generally provide more generous benefits than services received outside the network. |
| Health Maintenance Organizations (HMOs) | This type of plan assumes both the financial risks associated with providing comprehensive medical services and the responsibility for delivering health care in a particular geographic area, usually in return for a fixed, prepaid fee from members. Traditional HMOs provide no benefits for services obtained outside the network. Open-access HMOs allow enrollees to receive services from a non-network provider at a higher cost than the enrollee would pay at a network provider. |
| High deductible health plan (HDHP) | This type of plan typically features a higher deductible and lower insurance premiums than those of traditional health plans. The plan includes catastrophic coverage to protect against large medical expenses, but the insured is responsible for routine out-of-pocket expenses up until they meet the plan deductible. |
| Consumer-driven health plan (CDHP) | This type of plan combines a high-deductible health policy that provides protection from catastrophic medical expenses with a tax-favored account that pays routine health care expenses such as those for prescription medications and doctor visits. |

CDHP = Consumer-driven health plan; EPO = Exclusive provider organization; HDHP = High deductible health plan;
HMO = Health Maintenance Organizations; POS = Point-of-service; PPO = Preferred provider organization

1. United States Department of Labour. National Compensation Survey: Glossary of Employee Benefit Terms. 2017; <https://www.bls.gov/ncs/ebs/glossary20162017.htm#health_care>. Accessed June 28, 2018.
